# Supplementary material for: Identification and functional analysis of non-coding regulatory small RNA FenSr3 in Bacillus amyloliquefaciens LPB-18
Source: PeerJ. 2023 May 15;11:e15236. doi: 10.7717/peerj.15236 (PMC10194069; doi:10.7717/peerj.15236)
Supplement: Supplemental Information 4 [file peerj-11-15236-s004.zip › KO/CK-vs-T1_map/map05224.html]

KEGG PATHWAY: Breast cancer - Reference pathway


|  |  |
| --- | --- |
| **Breast cancer - Reference pathway** |  |

[
Pathway menu
| Organism menu
| Pathway entry
| Show description
| User data mapping
]

Breast cancer is the leading cause of cancer death among women worldwide. The vast majority of breast cancers are carcinomas that originate from cells lining the milk-forming ducts of the mammary gland. The molecular subtypes of breast cancer, which are based on the presence or absence of hormone receptors (estrogen and progesterone subtypes) and human epidermal growth factor receptor-2 (HER2), include: hormone receptor positive and HER2 negative (luminal A subtype), hormone receptor positive and HER2 positive (luminal B subtype), hormone receptor negative and HER2 positive (HER2 positive), and hormone receptor negative and HER2 negative (basal-like or triple-negative breast cancers (TNBCs)). Hormone receptor positive breast cancers are largely driven by the estrogen/ER pathway. In HER2 positive breast tumours, HER2 activates the PI3K/AKT and the RAS/RAF/MAPK pathways, and stimulate cell growth, survival and differentiation. In patients suffering from TNBC, the deregulation of various signalling pathways (Notch and Wnt/beta-catenin), EGFR protein have been confirmed. In the case of breast cancer only 8% of all cancers are hereditary, a phenomenon linked to genetic changes in BRCA1 or BRCA2. Somatic mutations in only three genes (TP53, PIK3CA and GATA3) occurred at >10% incidence across all breast cancers.

|  |  |  |
| --- | --- | --- |
| Reference pathway | 184% 150% 122% 100% 82% 67% 55% | 图片下载 |
